# Origin of gall-inducing from leaf-mining in *Caloptilia* micromoths (Lepidoptera, Gracillariidae)

**DOI:** 10.1038/s41598-019-43213-7

**Published:** 2019-05-01

**Authors:** Antoine Guiguet, Issei Ohshima, Seiji Takeda, Françoise Laurans, Carlos Lopez-Vaamonde, David Giron

**Affiliations:** 1 Institut de Recherche sur la Biologie de l’Insecte, UMR 7261, CNRS/Université de Tours, UFR Sciences et Techniques, Tours, France; 2 Department of Life and Environmental Sciences, Kyoto Prefectural University, Kyoto, Japan; 3 Cell and Genome Biology, Graduate School of Life and Environmental Sciences, Kyoto Prefectural University, Kyoto, Japan; 4 BioForA, INRA, ONF, 45075 Orléans, France; 5 INRA, UR0633 Zoologie Forestière, Orléans, France

**Keywords:** Evolutionary ecology, Evolutionary ecology, Evolutionary ecology, Evolutionary ecology, Entomology

## Abstract

In insects, the gall-inducing life-style has evolved independently many times. Several evolutionary pathways leading to this lifestyle have been proposed. While there is compelling evidence supporting surface-feeders and stem-borers as ancestral states of insect gall-inducers, an evolutionary pathway from leaf-miners remains hypothetical. Here we explored this question by comparing the developmental processes of two micromoths, a gall-inducer *Caloptilia cecidophora* (Lep., Gracillariidae), and its non-gall-inducing relative *C*. *ryukyuensis*. Like other *Caloptilia*, the first and second instars of *C*. *cecidophora* are leaf-miners and the gall is initiated inside the leaf mine by the third instar, thus suggesting leaf-mining as an ancestral, plesiomorphic state in this case. This is the first example of an insect species switching from leaf-mining to gall-inducing during larval development. The first two leaf-mining instars of *C*. *cecidophora* exhibit an absence of growth and a reduced time duration compared to *C*. *ryukyuensis*. The shortening of the duration of leaf-mining stages is apparently compensated in *C*. *cecidophora* by a larger egg size than *C*. *ryukyuensis*, and an additional larval instar during the gall phase.

## Introduction

In the class Insecta, the gall-inducing habit has evolved many times independently in six orders and is especially common among Hymenoptera, Diptera and Hemiptera^1^. Gall induction is defined as a plant growth response to stimuli produced by an inducing organism, generated through cell growth (hyperplasia) and/or cell enlargement (hypertrophy), and resulting in a phenotype specific to the gall-inducer species^2^. The phenotypic specificity distinguishes gall induction from non-specific foreigner-induced plant growth responses such as callus formation^3^. It has been hypothesized that the gall-inducing habit evolved either from the sedentary surface feeding habit or from stem boring^4^. Research efforts have been concentrated on the ‘sedentary’ route, where it has been shown that sawflies^5^, thrips^6^ and aphids^7^ that induce galls may have evolved from sedentary surface feeders. Regarding the other scenario, origins from the stem-boring habit, tephritid flies and in some caterpillars are proposed as examples^4^.

In addition to those two evolutionary pathways, we propose a third whereby gall induction evolved from the leaf-mining habit. Finding transitional states having gall-inducing characters in leaf-miner groups would support this third hypothesis. One such gall-inducing character is hyperplasia, which has been reported in some leaf mines^8,9^. In addition to the low number of cells generally produced, the nutritive role of the newly formed tissue for the insect and the role of the insect in its formation is debated^8,9^. Recently, we established the nutritive role of callus present in the leaf-mines of the micromoth *Borboryctis euryae* (Lep., Gracillariidae) and the active role played by the insect in the induction of this phenotype^3^. However, given that callus cells form an undifferentiated tissue, this induced phenotype cannot be considered as a phenotype specific to the gall-inducer species. Therefore, the existence of callus in leaf mines on its own cannot be considered as evidence that leaf-mining is an ancestral state of gall induction.

**Figure 1:**
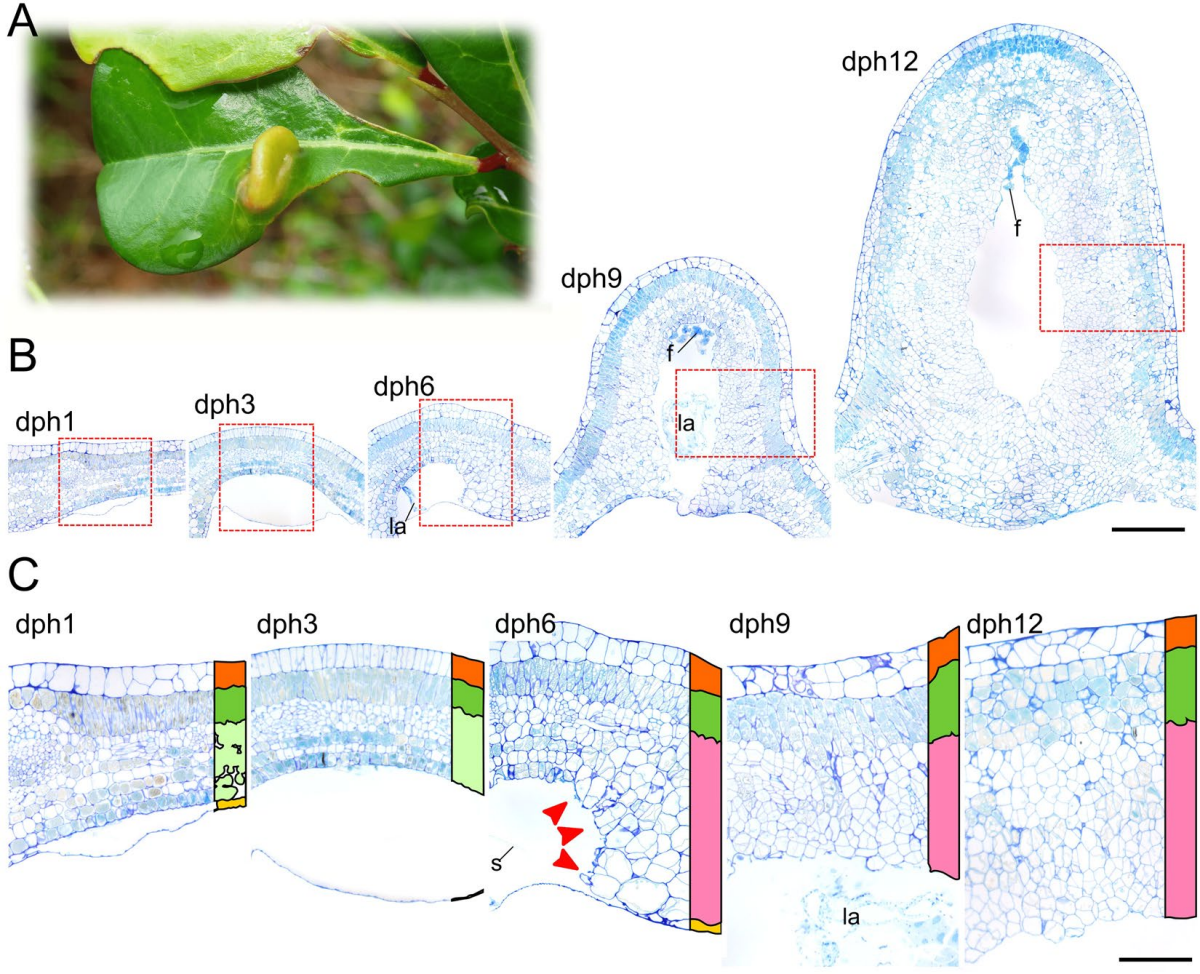
Gall initiated on *Glochidion obovatum* leaves attacked by *Caloptilia cecidophora* (**A**). Time series of histological sections of *C*. *cecidophora* gall initiation. (**B**) Cross sections of *G*. *obovatum* leaves attacked by *C*. *cecidophora* were made at 1 day post-hatching (dph1), at dph3, at dph6, at dph9, and at dph12. Red rectangles correspond to the zone detailed in (**C**). Legend, orange: upper epidermis, green: palisade parenchyma, light green: spongy parenchyma, yellow: lower epidermis, pink: altered parenchyma showing hypertrophied cells and an activation of cell division, s: silk, la: larvae, f: frass. Red arrows point cell division and hypertrophia orientated in direction to insect chamber. Scale: 500 µm for (**B**), 100 µm for (**C**). Staining: Toluidine Blue O.

Here, we explore the habits of the East Asian micromoth *Caloptilia cecidophora* (Lep., Gracillariidae) whose larva induces galls on *Glochidion obovatum*, *G*. *rubrum* and *G*. *acuminatum* (Phyllanthaceae) (Fig. 1). In the *Caloptilia* clade that feed on Phyllanthaceae, *C*. *cecidophora* is the only species that has been described as gall-inducer^10^. *Caloptilia* larvae typically have four successive styles of leaf-mining during larval development. Serpentine mines are created by first and early second instars, blotch mines by late second instars and three-dimensional tentiform mine by third instar. The remaining larval instars leave the mine and create a shelter by forming a leaf-roll^11^ (Fig. S1). In *C*. *cecidophora*, however, late instar larvae make a gall instead of a leaf-roll, and it has been suggested that early larval stages of *C*. *cecidophora* might be leaf-miners^10^.

We compared *C*. *cecidophora* larval development with that of its most closely related non-gall-inducing species, *C*. *ryukyuensis*. We propose that a gall-induction inside the mine provides support for direct evolution from leaf-mining, whereas a gall induction outside of the mine, which requires departure of the larva from the leaf mine gallery, provides support for the external feeding life-style being the ancestral state. To test these ideas, we established a time series of the histological changes that occur in mined leaves during the early stages of larval development. We aborted young larvae to test whether gall symptoms would appear in their absence. In addition, we compared the size of *C*. *cecidophora* and *C*. *ryukyuensis* at each developmental stage, in order to test the relationship between larval life-styles and patterns of larval growth.

## Methods

### Field collection and laboratory rearing

*Caloptilia cecidophora* galls on *Glochidion obovatum* were collected in Tomogashima Island (Wakayama, Japan; 34.28 N, 135.00 E) and maintained in a laboratory growth chamber (25 °C, 75% humidity, LP-1PH, NK system, Osaka, Japan) at Kyoto Prefectural University in Kyoto, Japan. Adults were sexed and transferred to mesh cages (40 × 40 × 40 cm) held in a greenhouse (25 °C, 70 ± 10% humidity). Each cage contained one or two pairs of moths and tissue paper soaked with a 2% sucrose solution, which was provided as a food for adults^12^. A branch of a potted *G*. *obovatum* bearing young leaves was placed in the cage. *C*. *cecidophora* females will only lay eggs on very fresh leaves. Oviposition was checked daily. When eggs were found, the twig was replaced. All rearing was conducted at Kyoto Prefectural University, Japan.

We also collected mines of a closely related^13^ non-gall-inducing species, *C*. *ryukyuensis*, on *G*. *zeylanicum* and *G*. *lanceolatum* in Ishigaki Island (Okinawa, Japan; 24.32-51 N, 124.07-27 E) and reared them on *G*. *zeylanicum* in Kyoto Prefectural University using the same protocol described above for *C*. *cecidophora*.

### Histological analysis of gall ontology

We made a time series of the initial stages of the gall development by sampling attacked leaves every three days, starting when the egg hatched and continuing until the gall with its larval chamber had been formed. To analyze the internal structure of a mature gall, we sectioned field collected galls occupied by fifth instars. To test whether galls are induced by third instar larvae, we killed second instar larvae three days post hatching (dph3) and then sampled the leaf at dph6. We killed the larvae with a thin needle. Larval instars were determined by counting the number of head-capsule moults of previous instars^14,15^. Head capsules were collected inside the leaf mines and identified by binocular microscope and transmitted light.

Cross-sections (about 2 mm wide) were cut with a surgical scalpel and fixed for 4 h with 2.5% paraformaldehyde and 0.4% glutaraldehyde in 0.1 M McIlvaine citrate-phosphate buffer, pH 7.0. After dehydration in a graded series of ethanol, samples were embedded in medium grade LR White resin (London Resin Company Ltd, UK). Semi-thin sections (1 µm thick) were cut with a diamond knife (Diatome, Biel, Switzerland), or a triangular glass knife for the experiment in which the larva has been removed, installed in an ultracut R microtome (Leica, Rueil-Malmaison, France), placed on slides, fixed by heating at 120 °C for 2 min and stained with Toluidine Blue O 0,1% (w/v in water) (Sigma-Aldrich, T0394). Images were assembled using MosaicJ plugin of ImageJ^16^. Photoshop (Adobe) was used to adjust contrast and white balance, remove background, and for cropping.

### Assessment of insect development

We compared the immature growth patterns of *C*. *cecidophora* with its closely related non-gall-inducing species, *C*. *ryukyuensis*, by measuring eggs (N_*C*. *cecidophora*_ = 20 N_*C*. *ryukyuensis*_ = 11), larvae (N comprised between 6 and 22, for details see Table S8) and pupae (N_*C*. *cecidophora*_ = 10 N_*C*. *ryukyuensis*_ = 7). After inducing oviposition of field-collected pairs of both species, we measured egg width and length. After hatching, we measured head-capsule width of first and second instars. Later larval instars and pupae of *C*. *cecidophora* and *C*. *ryukyuensis* were directly collected from the field (Tomogashima and Ishigaki Islands, respectively). We measured head-capsule with the micrometer of a light microscope (Leica M205C). In addition, we measured the duration of both first and second instars and filmed each stage of larval development for both species (Pentax K3, with binocular microscope Leica S6E).

The shape of the egg area was approximated being an ellipse. Area was calculated using length and width measurements. Egg areas, head-capsule widths and pupa lengths were compared using Welch’s *t*-tests. For the comparisons of larval head capsules among instars, we applied the sequential Bonferroni correction^17^ to *p*-values to keep the significance level at 0.05 throughout the tests. The corrected *p*-values are indicated as *p* (*adjusted*). All statistical analyses were conducted using RStudio version 1.1.453 (RStudio, Boston, USA) with R 3.3.1 (R Developmental Core Team 2016).

Larval head morphology before and after gall induction began, in particular mouthparts of the second and third instars of *C*. *cecidophora*, was analyzed using scanning electron microscopy. Larvae were dehydrated in ethanol overnight, mounted on scanning electron microscopy stubs, coated with gold, and observed with a scanning electron microscope (JEOL DATUM, JSM-5800LV) at an accelerating voltage of 20 kV. Images were assembled using MosaicJ plugin of ImageJ. Colors were added using Inkscape (v0.92, www.inkscape.org).

## Results

### Gall ontology

First and second instars of *C*. *cecidophora* mine along the lower side of the leaf (Fig. S1, Movie S2). This is also the case for *C*. *ryukyuensis* and other *Caloptilia* (Fig. S1, Movie S3). Analysis of the time series of cross-sections of attacked leaves (Fig. 1) revealed that the first instar makes a gallery inside the lower epidermis at one day-post hatching (dph1). This mine is enlarged into a blotch mine by the second instar, which begins dph3. The absence of any tissue alteration shows that those two instars are true leaf-miners.

The third instar begins at dph4. Two days later, at dph6, the mine induced by *C*. *cecidophora* differs from the types of mines induced by other *Caloptilia* species by the formation of hypertrophied cells showing periclinal divisions (i.e. cell divisions whose division plan is parallel to the leaf surface) in the spongy parenchyma located at the edges of the mine (Fig. 1). Cell hypertrophy and hyperplasia - activation of cell divisions - occurred after the insect reaches the third instar. This evidence suggests that gall induction is initiated by the third instar. Such tissue alterations are not observed in third instar mines of *C*. *ryukyuensis*. (Fig. S5).

**Figure 2:**
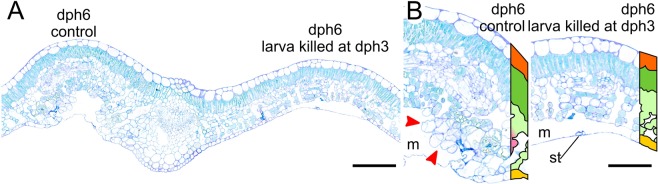
Histological sections of *Caloptilia cecidophora* gall after larva abortion. Cross sections of *Glochidion obovatum* leaves attacked by *C*. *cecidophora* were made at 6 day post-hatching (dph6) after killing the larva at dph3. Legend, m: mine, st: stomata, for remaining see Fig. 1. Scale: 500 µm for (**A**), 100 µm for (**B**). Staining: Toluidine Blue O.

To test whether gall induction coincides with the beginning of the third instar, we killed the late second instar larva and observed the effect on the development of the leaf region attacked by the insect. Three days after killing the late second instar larva, no alteration was observed in the spongy parenchyma cells adjacent to the larva, contrary to the control leaf-mine in which the same tissue exhibits cell hyperplasia and hypertrophy (Fig. 2), demonstrating that gall induction requires the third instar larva.

**Figure 3:**
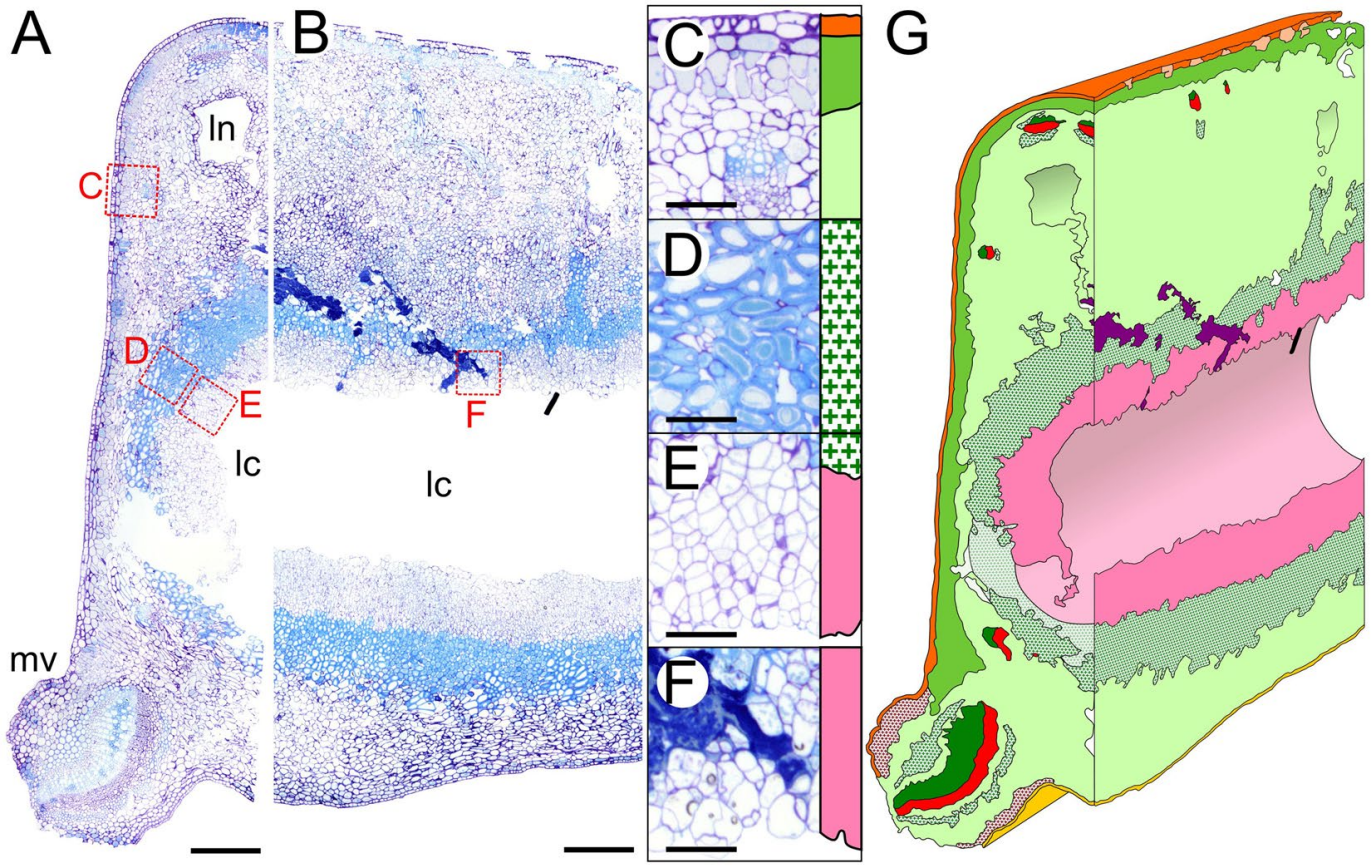
Histological sections and 3D modeling of *Caloptilia cecidophora* gall at late larval instar. (**A**) Transversal section. (**B**) Sagittal section. (**C**) Detail of gall epidermis. (**D**) Detail of the sclerenchyma. (**E**) Detail of nutritive tissue. (**F**) Detail of insect frass trapped in nutritive tissue. (**G**) 3D modeling of a mature gall based on histology sections. Legend, dark green: xylem, red: phloem, pink: nutritive tissue, green cross: scerenchyma, red cross: colenchyma, purple: frass, lc: larva chamber, ln: lacuna, mv: midvein, for remaining see Fig. 1. Scale: 500 µm for (**A**,**B**), 100 µm from (**C**–**F**). Staining: Toluidine Blue O.

The closure of the gall at the lower side starts with third instar using silk to fold up the leaf cuticle of the lower epidermis (Movie S2). Gall closure is completed at dph12 by the coming together of the newly formed tissues (Fig. 1). Frass accumulates in the upper region of the gallery from dph9. The presence of frass within the tissue of the same region at latter stage of gall development (Fig. 3) provides evidence that the dense parenchyma that lines the larva merges also at the upper part to form an insect chamber.

In mature galls (Fig. 3), the insect chamber is lined with two kinds of tissues that are absent in the normal leaf: a sclerenchyma, and a dense parenchyma that presents traces of feeding. In addition, we found an antero-posterior asymmetry in the sagittal section of mature galls (Fig. S6). Sclerenchyma is absent in the anterior part but present in the posterior, where it co-occurs with frass. At the end of larval development and before pupation, the dense parenchyma is entirely eaten in the anterior region of the gall. A “window” of intact upper epidermis cuticle through which the adult moth escapes is appeared at the anterior surface (Fig. S7).

### Larval development

**Figure 4:**
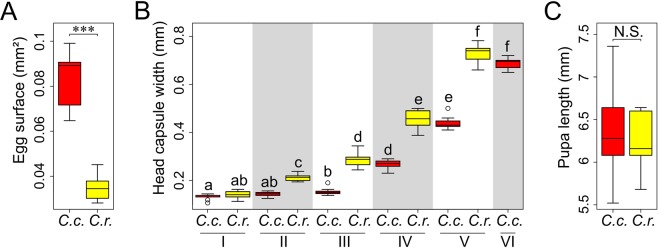
Boxplots showing insect size along development of *Caloptilia cecidophora* (*C*.*c*., in red) and its closely related non-gall-inducing species *C*. *ryukyuensis*. (*C*.*r*., in yellow) (**A**) Egg area. (**B**) Head capsule width of each larva instar, instars followed by a different letter have significantly different size (see Table S8). (**C**) Pupal length (***<0.001, N.S.: no significant difference).

It took three days for *C*. *cecidophora* larvae to moult to the third instar (n = 8), whereas *C*. *ryukyuensis* took five days (n = 9). Eggs of *C*. *ryukyuensis* are smaller than those of *C*. *cecidophora* (*t* = 16.075, df = 28.542, *p*-value = 7.72e-16) (Fig. 4A). The first larval instars of both species have similar head capsule widths (Fig. 4B, Table S8). *C*. *ryukyuensis* larval body size increases significantly at each instar, whereas the body size of *C*. *cecidophora* larvae remains the same through the third instar (Fig. 4B). Larval size starts to increase at the fourth instar. It continues to grow during the fifth instar. *C*. *cecidophora* possess a sixth instar, an additional developmental stage in comparison with the five larval instars of *C*. *ryukyuensis* and other *Caloptilia*^18^. This extra sixth instar shows similar head capsule width as the fifth instar of *C*. *ryukyuensis*. Pupae of both species show similar body length (*t* = 0.37783, df = 14.996, *p*-value = 0.7109).

In both species, the head morphology of the second larval instar is as follows: (1) flat prognathous head, (2) maxillae, labial palpi and (3) spinneret are absent and mandibles are flattened plates (Movies S2 and S3, Fig. S4). These features are consistent with fluid-feeding behaviour in caterpillars^19^. In both species, third instar larvae show mandibles that conform to the normal lepidopteran chewing type (Movies S2 and S3, Fig. S4). This indicates that larval hypermetamorphosis in *C*. *cecidophora* occurs between the second and third instar, as in many other *Caloptilia* species^14^, but in this case, happens concurrently with the switch to feeding on induced gall tissue.

## Discussion

Our results establish the existence of a leaf-mining stage during the first two larval instars of the gall-inducing micromoth *C*. *cecidophora*. This is the first example of an insect switching from leaf-mining to the induction of a complex gall. Gall-like structures have previously been described in leaf-mines but differ from what are described here because they only involve a limited formation of callus^3,8^. Our results provide evidence for a transitional state between leaf-miners and gall-inducers. This suggests that leaf-miners offers a third evolutionary pathway to the gall-inducing habits of insects. Price previously proposed two evolutionary pathways, one from sedentary herbivory and another from boring herbivory^10^.

**Figure 5:**
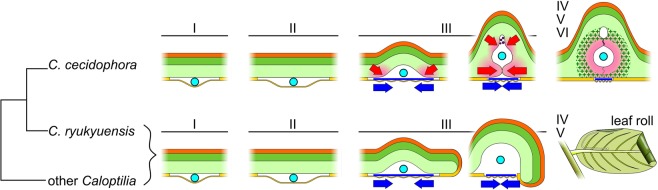
*Caloptilia cecidophora* interaction with its host plant compared to *C*. *ryukyuensis* and other *Caloptilia*. Every first and second instars are epidermis miners in this genus. Like other *Caloptilia*, *C*. *cecidophora* third instar folds its bloch mine into a tentiform shape by weaving silk threats on lower epidermis; then, the mine folding is the result of mechanical forces (blue arrows). However, contrary to other species of the genus, *C*. *cecidophora* activates tissue growth at its feeding sites, inducing morphogenetic movements (red arrows) that progressively seal the two joined upper leaf sides. Secondary morphogenesis occurs at upper part of the larval chamber, trapping frass and creating a lacuna. Late stages of this species stay in the gall until pupation, whereas other *Caloptilia* leave the tentiform mine to build a leaf-roll.

We show that the gall stage evolves from a tentiform mine and that the induction of the gall occurs at the third instar (Fig. 5). Like most other *Caloptilia* species, the third instar larva draws together the spongy parenchyma of the mine edges using silk (Movie S2). However, in the case of *C*. *cecidophora*, activation of cell division at the mine edges leads to their fusion, which progressively creates a closed cavity inside the altered spongy parenchyma. The gall closure mechanism is a mix of a physical process, the lower epidermis folding involving the use of silk, and an uncharacterized chemical process, activation by the larva of leaf cell hypertrophy and proliferation through hyperplasia (Fig. 5). To our knowledge, a gall ontology that begins with mechanical folding of the leaf by the insect has never been described before in arthropod-induced galls. Furthermore, this shows that folding of a three-dimensional tentiform mine might constitute a preadaptation to gall induction, thus supporting that induction of leaf depression can precede leaf gall evolution^10^.

The gall-inducing habit is associated with a significant reduction in the duration of the leaf-mining larval stage. In *C*. *cecidophora* the first two leaf-mining instars last only three days compared to five days for its closest relative *C*. *ryukyuensis*. The larva of *C*. *ryukyuensis* builds what appears to be a longer serpentine mine in the first instar. In contrast, the larva of *C*. *cecidophora* builds a mine whose dimensions are reduced to what appears to be the minimal surface required for the creation of a tentiform mine.

In addition, this shortening of early larval development duration noticed in *C*. *cecidophora* is associated with a difference of growth pattern in comparison to *C*. *ryukyuensis* (Fig. 4). Whereas *C*. *ryukyuensis* has a continuous growth, *C*. *cecidophora* larvae keep the same size from the first to the third instar. *C*. *cecidophora* larval growth begins when the gall starts to develop. A similar pattern is seen in gall inducing cynipids, for which larvae remain very small until putatively defensive gall morphologies have developed^20^. As a result, the larval growth accumulates a one-instar delay compared to *C*. *ryukyuensis*. *C*. *cecidophora* makes up for this early absence of growth by adding a sixth instar that does not occur in other *Caloptilia* species. This peculiar growth pattern compared to its non-gall-inducing sister species suggests an intense selective pressure to reduce the duration of the early leaf-mining stages. It supports the hypothesis that early stages of gall formation, when the insect does not benefit yet from gall protection, represent a “window of vulnerability” to enemy attacks^21–23^.

The internal structure of *C*. *cecidopho*ra galls with a layer of neovascularized nutritive tissue surrounded by sclerenchyma (Fig. 3), which confers mechanical resistance to the gall shows the ability of *C*. *cecidophora* to induce tissue differentiation. This internal organization in two layers is similar to some of the most complex galls of cynipids or cecidomyiids^20^. This convergent internal structure suggests that there might be limited ways of inducing galls due to plant physiological constraints.

Gall induction did not proceed after we killed the second instar leaf-mining larva. This suggests that the cecidogenous substance is only secreted from the third larval instar. This differs from other gall inducers for which gall induction begins at the start of insect’s interaction with the host plant either as a feeding larva (e.g. cecidomyiids and aphid) or as an ovipositing adult (e.g. cynipids). This particularity may represent an opportunity to better understand the mechanism of gall induction and to identify chemical effectors involved. Omics and chemical investigations are facilitated by comparative approaches^24^. The mixed feeding habit of *C*. *cecidophora* and its phylogenetic proximity with non-gall-inducing relatives allow such approaches. In addition, we developed a rearing protocol to produce large number of adults from eggs in greenhouses. These lab colonies can be used to set up experiments in controlled conditions, making *C*. *cecidophora* an excellent model to investigate fundamental questions about the physiology and evolution of gall induction.

## Supplementary information

Supplementary information

Movie S2

Movie S3
