# Supplementary material for: Origin of gall-inducing from leaf-mining in Caloptilia micromoths (Lepidoptera, Gracillariidae)
Source: Sci Rep. 2019 May 1;9:6794. doi: 10.1038/s41598-019-43213-7 (PMC6494848; doi:10.1038/s41598-019-43213-7)
Supplement: Supplementary file 1 — Supplementary information [file 41598_2019_43213_MOESM1_ESM.pdf]

<u>Supplementary information</u>

Origin of gall-inducing from leaf-mining in *Caloptilia* micromoths (Lepidoptera, Gracillariidae)

Antoine Guiguet, Issei Ohshima, Seiji Takeda, Françoise Laurans, Carlos Lopez-Vaamonde, David Giron

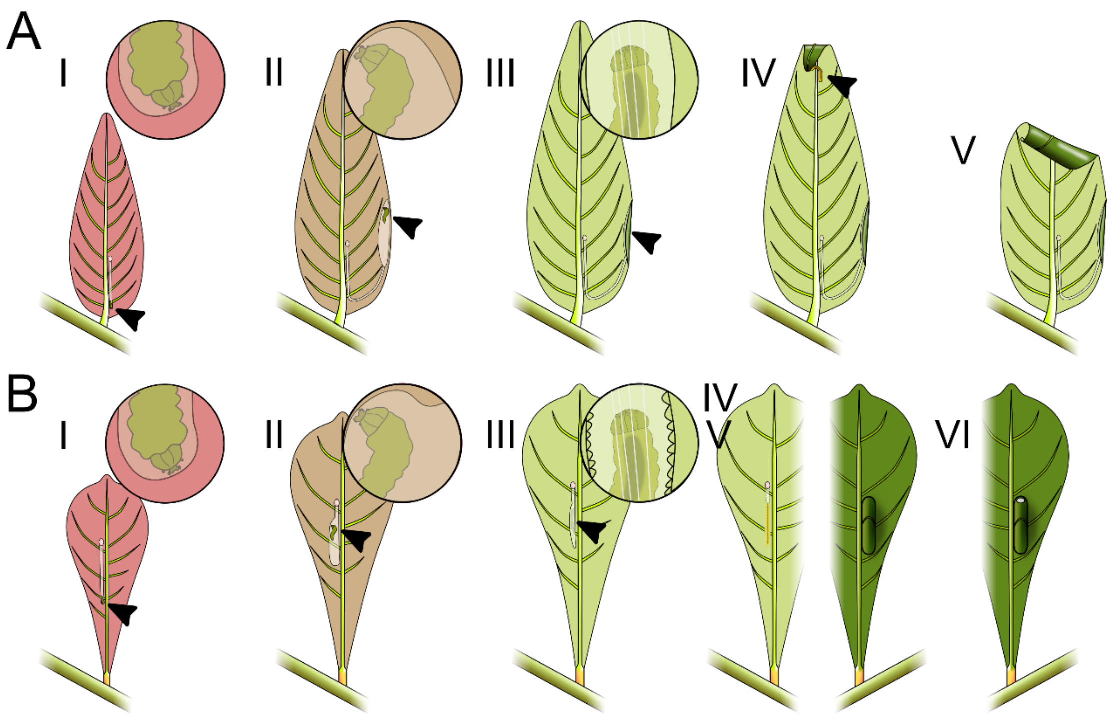

Figure S1.

Caloptilia ryukyuensis life-style (**A**) compared to *Caloptilia cecidophora* life-style (**B**). Both species make a serpentine mine at first instar, a blotch mine at second instar and a tentiform mine at third instar. *C. cecidophora* starts to induce a gall inside this mine and will stay inside it until sixth instar and pupation, whereas *C. ryukyuensis* leaves its mine at fourth instar to build a leaf-roll in which it will complete its larval development.

Movie S2.

Movie showing *Caloptilia cecidophora* development on *Glochidion obovatum*.

Movie S3.

Movie showing *Caloptilia ryukyuensis* development on *Glochidion lanceolatum*.

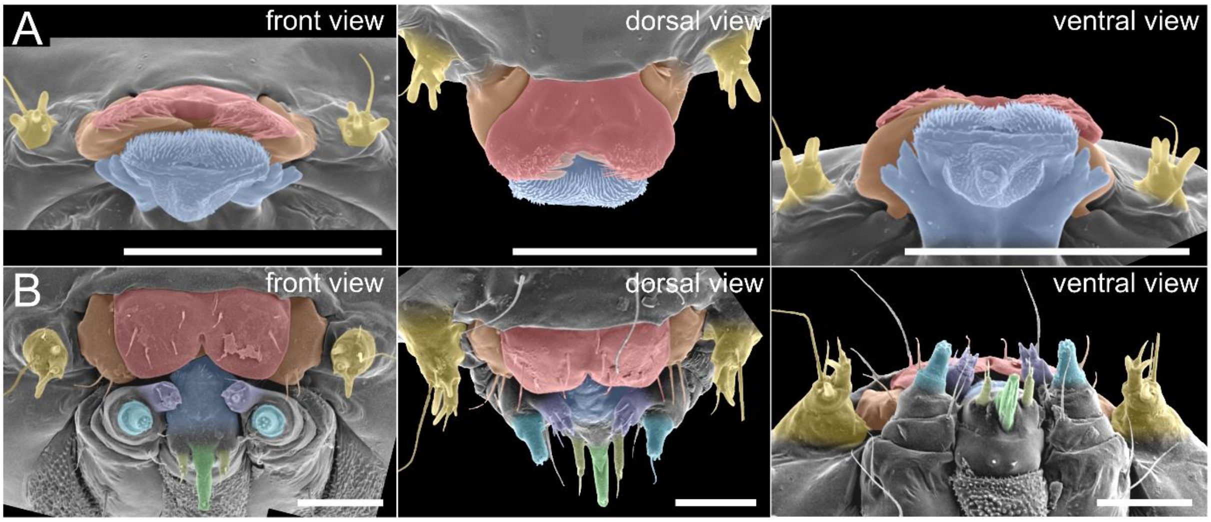

Figure S4.

Mouthparts of *Caloptilia cecidophora* second (**A**) and third instars (**B**) as seen in scanning electron microscopy. Legend, red: labrum, yellow: antenna, orange: mandibles, purple: galea, dark blue: labium, light blue: maxillar palpi, dark green: labial palpi, light green: spinneret. Scale: 100µm.

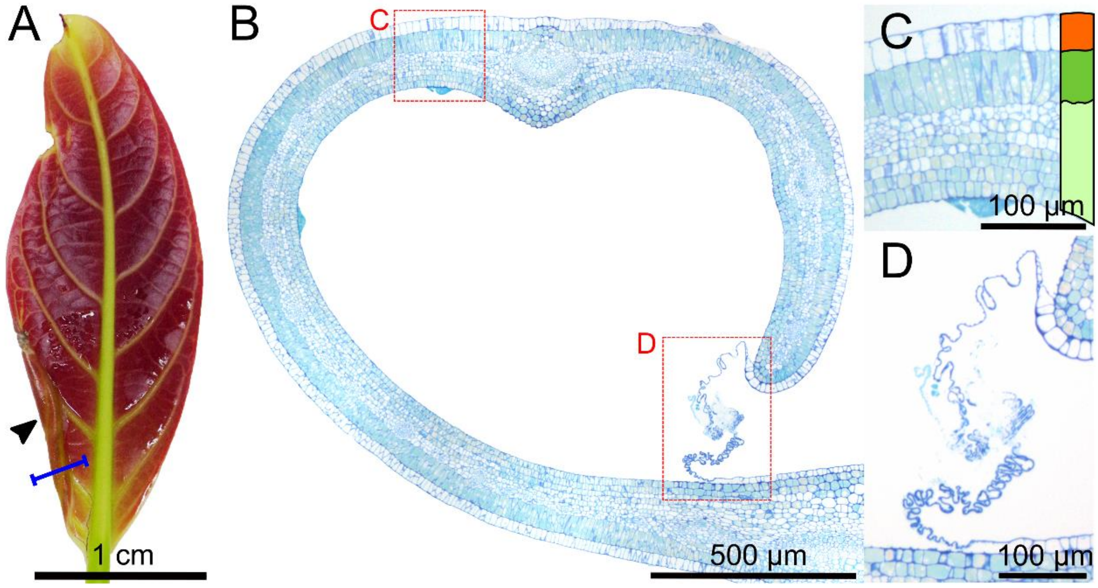

Figure S5.

Tentiform mine of *Caloptilia ryukyuensis* third instar, indicated with black arrow on *G. lanceolatum* (**A**). Blue line indicates the region cross-sectioned. (**B**) Histology section of the tentiform mine. (**C**) Detail of mined leaf. (**D**) Detail of folded upper epidermis cuticle. Legend, see figure 1. Staining: Toluidine blue.

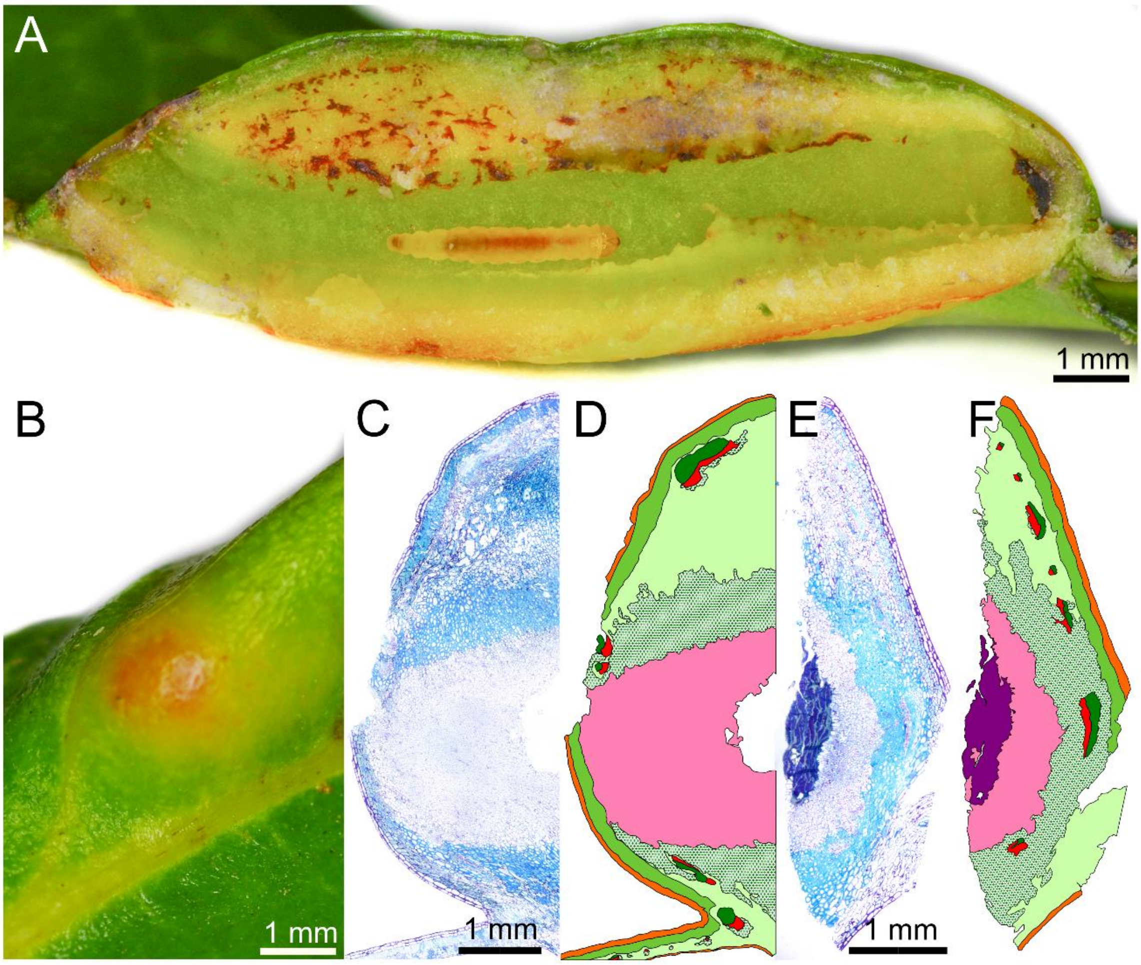

Figure S6.

(**A**) Sagittal section of *Caloptilia cecidophora* late instar gall on *Glochidion obovatum*. (**B**) Detail of the anterior part of the gall. (**C**) Histology sagittal section of the anterior part and (**D**) the interpretation drawing. (**E**) Histology sagittal section of the posterior part and (**F**) the interpretation drawing. Legend, see figure 1 and 3.

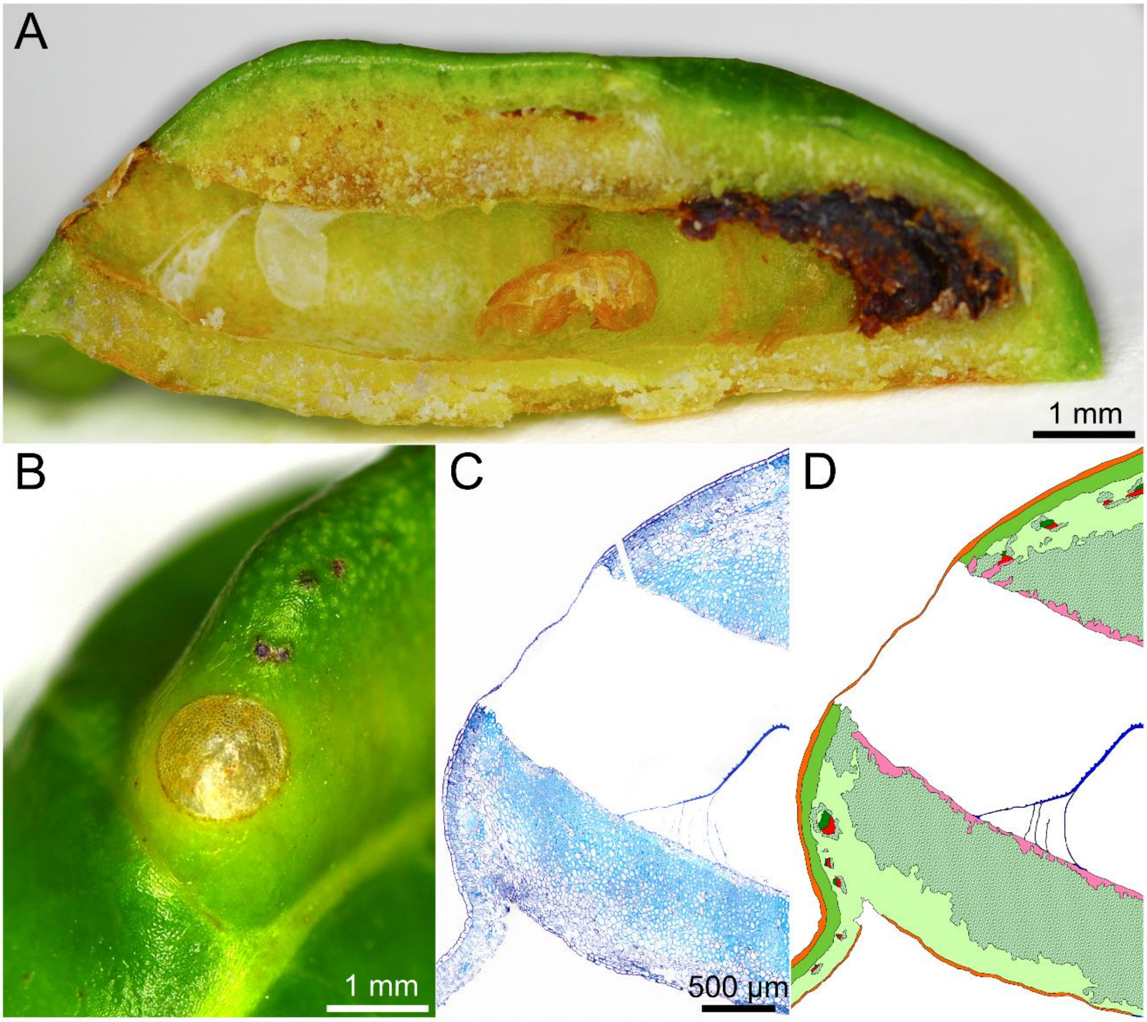

Figure S7.

(**A**) Sagittal section of *Caloptilia cecidophora* gall at pupa stage on *Glochidion obovatum*. (**B**) Detail of the anterior part of the gall. (**C**) Histology sagittal section of the anterior part and (**D**) the interpretation drawing. Legend, dark blue: silk, for remaining see figure 1 and 3.

Table S8.

Welch’s t-test statistics for the comparison of *Caloptilia cecidophora* (Cc) and *C. ryukyuensis* (Cr) head capsule widths. Numbers indicate the instars. Bold *p*-values are smaller than

N	*p* \ *t*	Cc1	Cr1	Cc2	Cr2	Cc3	Cr3	Cc4	Cr4	Cc5	Cr5	Cc6
9	Cc1		-1.1385	-2.1398	-15.217	-4.2468	-19.795	-23.81	-34.065	-35.752	-54.05	-50.749
8	Cr1	0.2752		-0.41456	-10.966	-1.9745	-16.775	-18.445	-30.768	-31.747	-50.249	-47.163
12	Cc2	0.0499	0.687		-16.495	-2.6701	-20.142	-26.365	-34.889	-37.056	-55.398	-51.938
14	Cr2	**4.00E-11**	**2.17E-07**	**1.44E-14**		15.248	-10.218	-11.136	-26.86	-27.976	-48.316	-44.928
22	Cc3	**0.00094**	0.07861	0.01308	**2.05E-14**		-19.243	-25.701	-34.319	-36.522	-55.063	-51.566
16	Cr3	**8.76E-16**	**1.46E-13**	**1.13E-14**	**1.09E-09**	**9.40E-14**		2.522	-15.864	-15.468	-36.33	-33.412
20	Cc4	**2.20E-16**	**4.48E-11**	**2.20E-16**	**1.53E-12**	**2.20E-16**	0.0185		-20.266	-20.528	-42.244	-38.954
18	Cr4	**2.20E-16**	**2.20E-16**	**2.20E-16**	**2.20E-16**	**2.20E-16**	**2.35E-16**	**2.20E-16**		1.455	-20.109	-17.513
11	Cc5	**4.16E-16**	**2.20E-16**	**3.16E-14**	**2.56E-13**	**1.52E-13**	**2.09E-13**	**1.59E-12**	0.1574		-22.446	-19.709
10	Cr5	**1.32E-15**	**2.20E-16**	**4.26E-14**	**6.40E-14**	**1.15E-13**	**2.20E-16**	**4.27E-14**	**3.88E-15**	**4.25E-14**		2.3539
6	Cc6	**6.66E-10**	**4.02E-11**	**6.99E-09**	**8.58E-09**	**1.31E-08**	**3.88E-11**	**6.34E-09**	**2.72E-10**	**1.35E-09**	0.03522	

p(adjusted))
